# Quantitative Microbial Risk Assessment of *Listeria monocytogenes* and Enterohemorrhagic *Escherichia coli* in Yogurt

**DOI:** 10.3390/foods11070971

**Published:** 2022-03-27

**Authors:** So Young Yang, Ki Sun Yoon

**Affiliations:** Department of Food and Nutrition, College of Human Ecology, Kyung Hee University, 26 Kyungheedae-ro, Dongdaemun-gu, Seoul 02447, Korea; sy2ang@khu.ac.kr

**Keywords:** *Listeria monocytogenes*, enterohemorrhagic *Escherichia coli*, yogurt, quantitative microbial risk assessment

## Abstract

*Listeria monocytogenes* can survive in yogurt stored at a refrigeration temperature. Enterohemorrhagic *Escherichia coli* (EHEC) has a strong acid resistance that can survive in the yogurt with a low pH. We estimated the risk of *L. monocytogenes* and EHEC due to yogurt consumption with @Risk. Predictive survival models for *L. monocytogenes* and EHEC in drinking and regular yogurt were developed at 4, 10, 17, 25, and 36 °C, and the survival of both pathogens in yogurt was predicted during distribution and storage at home. The average initial contamination level in drinking and regular yogurt was calculated to be −3.941 log CFU/g and −3.608 log CFU/g, respectively, and the contamination level of both LM and EHEC decreased in yogurt from the market to home. Mean values of the possibility of illness caused by EHEC were higher (drinking: 1.44 × 10^−8^; regular: 5.09 × 10^−9^) than *L. monocytogenes* (drinking: 1.91 × 10^−15^; regular: 2.87 × 10^−16^) in the susceptible population. Both pathogens had a positive correlation with the initial contamination level and consumption. These results show that the foodborne illness risk from *L. monocytogenes* and EHEC due to yogurt consumption is very low. However, controlling the initial contamination level of EHEC during yogurt manufacture should be emphasized.

## 1. Introduction

Yogurt is a dairy product fermented by *Streptococcus thermophilus* and *Lactobacillus bulgaricus* [1]. Yogurt provides probiotics known to be beneficial bacteria that can promote health. Worldwide, the consumption of probiotics and yogurt is increasing every year [2,3,4].

Pathogenic *Escherichia coli* (*E. coli*) are a group of facultative anaerobes that can cause diseases in healthy individuals with a combination of certain virulence factors, including adhesins, invasins, toxins, and capsules. Pathogenic *E. coli* are classified into six pathotypes based on clinical, epidemiological, and virulence traits: enteropathogenic *E. coli* (EPEC), enteroaggregative *E. coli* (EAEC), diffusely adherent *E. coli* (DAEC), enterotoxigenic *E. coli* (ETEC), enteroinvasive *E. coli* (EIEC) and enterohemorrhagic *E. coli* (EHEC) [5]. EPEC (60.5%) is the primary cause of pathogenic *E. coli* outbreaks in Korea, followed by ETEC (31.2%), EHEC (6.8%), and EIEC (1.5%) [6]. Among them, EHEC can cause diarrhea with a mechanism of attaching-effacing (A/E) lesions with only a low infectious dose (1–100 CFU) [7]. EHEC has strong acid resistance that can make it viable in food with a low pH [8]. Morgan et al. [9] reported 16 cases of *E. coli* O157:H7 Phage Type 49 due to the consumption of a locally produced yogurt occurring in the northwest of England in 1991. In a study by Cutrim et al. [10], *E. coli* O157:H7 was shown to survive for 10 days in both traditional inoculated yogurt and pre-hydrolyzed inoculated yogurt, whereas its survival increased to 22 days in lactose-free yogurt. The populations of *E. coli* O157:H7 decreased by only about 1.4 log CFU/g after 28 days in Greek-style yogurt [11].

*Listeria monocytogenes* (LM) are facultatively anaerobic opportunistic pathogens that can grow between 0 and 45 °C; optimal growth occurs at 30~37 °C [12]. It can grow at pH 4–9.6 [13]. Listeriosis is caused by LM, which can cross the intestinal barrier and spread to lymph and blood to reach target organs such as the liver and spleen. Moreover, LM can be fatal to immunocompromised individuals, newborns, older adults, and pregnant women since LM can penetrate the blood–brain barrier or the fetoplacental barrier [14,15]. The approximate infective dose of LM is estimated to be 10 to 100 million CFU in healthy hosts and only 0.1 to 10 million CFU in individuals at high risk of infection [16]. In the US, a significant number of LM outbreaks are caused by raw milk, unpasteurized milk, cheeses, and ice cream [17]. Improper management of pasteurization temperature or technical imperfections can lead to the contamination of dairy products [18].

Risk assessment can estimate the probability of occurrence and severity of adverse effects in humans exposed to foodborne hazards [19]. Quantitative microbial risk assessment (QMRA) provides numerical estimates of risk exposure to identify which factors affect the exposure [20]. QMRA consists of hazard identification, hazard characterization, exposure assessment, and risk characterization [19]. Hazard identification is the step that identifies the presence of microorganisms or microbial toxins in a particular food based on the scientific literature. In the hazard characterization step, it is possible to perform qualitative and quantitative assessments of the adverse effects of consuming food contaminated by microorganisms [21]. Exposure assessment is the process that characterizes the level of hazard exposed to the population [22]. The final step of QMRA is risk characterization that provides the possibility of illness/person/day of pathogens when consuming contaminated food [21]. A risk assessment study of *Staphylococcus aureus* in milk and homemade yogurt was reported in Ethiopia [23]. Results showed the importance of traditional food preparation methods, such as fermentation, in risk mitigation; yogurt, traditional milk fermentation, reduced the risk by 93.7%. QMRA of LM and enterohemorrhagic *E. coli* in yogurt has not been reported yet. Therefore, the objective of this study was to conduct a microbial risk assessment for *L. monocytogenes* and enterohemorrhagic *E. coli* to compare their risks in drinking and regular yogurt.

## 2. Materials and Methods

### 2.1. Prevalence and Initial Contamination Level in an Offline Market

To derive prevalence (PR) data of LM and EHEC in yogurt by season and location, results of yogurt monitoring (195 drinking yogurts and 90 regular yogurts) were used [24]. LM and EHEC were identified with methods as described in the Korean Food Code [25]. The distribution of PR was fitted using Beta distribution (α, β), with α meaning “number of positive samples+1” and β meaning “number of total samples-number of positive samples +1” [26]. Initial contamination levels of LM and EHEC were estimated using the equation [Log (-ln(1-PR)/weight)] of Sanaa et al. [27].

### 2.2. Physicochemical and Microbiological Analyses of Yogurt

Ten products of two types of yogurt (drinking and regular) were purchased from an offline market. The pH, water activity (Aw), total aerobic bacteria, coliform, and *E. coli* were measured. Briefly, 10 g of sample was aseptically placed in a stomacher bag with 90 mL of distilled water and homogenized with a stomacher (Interscience, Paris, France). The pH was measured with a pH meter (Orion^TM^ Star A211, ThermoFisher Scientific Co., Waltham, MA, USA). The Aw of each sample (15 g) was measured in triplicate using a water activity meter (Rotronic HP23-AW-A, Rotronic AG, Bassersdorf, Switzerland). To measure total aerobic bacteria (AC), coliform, and *E. coli* (EC), 25 g of sample was homogenized with 225 mL of 0.1% sterile peptone water (BD, Sparks, MD, USA) and serially diluted 10-fold with 0.1% peptone water. After inoculating 1 mL aliquot of each dilution onto two or more sheets of 3M Petrifilm *E. coli*/Coliform Count Plate (3M corporation, St. Paul, MN, USA), AC and EC plates were incubated at 36 ± 1 °C for 48 h and 24 h, respectively.

### 2.3. Strain Preparation

An LM strain isolated from the gloves of a slaughterhouse worker [28] was stored in tryptic soy broth (TSB, MB cell, Seoul, Korea) containing 0.6% yeast extract with 20% glycerol (Duksan, South Korea) at −80 °C. After thawing at ambient temperature, 10 μL of LM inoculum was added into 10 mL of TSB containing 0.6% yeast extract and then cultured at 36 ± 1 °C for 24 h in a 140 rpm rotary shaker (VS-8480, Vision Scientific, Daejeon, Korea).

*E. coli* (EHEC) strains (NCCP 13720, 13721) including *E. coli* O157:H7 (NCTC 12079) were obtained from the Ministry of Food and Drug Safety (MFDS) in Korea. After thawing frozen strains that were stored at −80 °C, they were cultured in the same way as described above. All strains were centrifuged at 4000 rpm for 10 min (VS-550, Vision Scientific, Daejeon, Korea) and the supernatant was removed. Pellets were harvested by centrifugation (4000 rpm for 10 min), washed with 10 mL of 0.1% peptone water, and resuspended with 0.1% peptone water to a final concentration of approximately 9.0 log CFU/mL.

### 2.4. Sample Preparation and Inoculation

For model development, the popularity of yogurt samples and results of physicochemical (high pH value) and microbiological analyses of yogurt were considered. Drinking and regular yogurt were purchased from an offline market (Seoul, Korea) and aseptically divided into 30 mL and 10 g, respectively, into 50 mL conical tubes (SPL Life Science Co., Daejeon, Seoul). LM and the cocktail of *E. coli* strains were independently inoculated into drinking (4~5 log CFU/g) and regular yogurts (5~6 log CFU/g). Each sample was then stored at 4, 10, 17, 25, and 36 °C until no colonies were detected for up to 21 days. At a specific time, each yogurt sample was homogenized with sterilized 0.1% peptone water for 120 s using a stomacher. Then 1 mL of the aliquot of the homogenate was serially diluted ten-fold with 0.1% peptone water and spread onto PALCAM agar (Oxoid, Basingstoke, Hampshire, UK) for LM and EMB agar (Oxoid, Basingstoke, Hampshire, UK) for EHEC, which were incubated at 36 ± 1 °C for 24 h to analyze the change in pathogen populations.

### 2.5. Development of Primary and Secondary Model

The Weibull model [29] (Equation (1)) and GinaFit V1.7 program [30] were used to develop the primary survival model of yogurt as a function of temperature. Delta value (time for the first decimal reduction) and *p*-value (shape of graph) were then calculated.

(1) $$\mathrm{Weibull}\;\mathrm{equation}:\;\mathrm{Log}\left(N\right)=\mathrm{Log}\left(N_{0}\right)-{\left(\frac{t}{delta}\right)}^{p}$$

*N*_0_: log initial number of cells

*t*: time

*delta*: time for the first decimal reduction

*p*: shape (*p* > 1: concave downward curve, *p* < 1: concave upward curve, *p* = 1: log-linear)

From results obtained through the primary predictive model, the secondary model was developed by applying the third-order polynomial model (Equation (2)) to delta values of both LM and EHEC as a function of temperature.

(2) $$\mathrm{Third}-\mathrm{order}\;\mathrm{polynomial}\;\mathrm{model}:\;Y=b_{0}+b_{1}\times T+b_{2}\times T^{2}+b_{3}\times T^{3}$$

Y: delta (d)

*b*_0_, *b*_1_, *b*_2_, *b*_3_: constant

*T*: temperature

### 2.6. Validation

To verify the applicability of the predictive model of LM, the delta value was obtained with temperatures not used for model development in this study, which was 7 °C for drinking yogurt and 13 °C for regular yogurt (interpolation). The predictive model of EHEC was verified with enteropathogenic (EPEC) strain (extrapolation), which was detected in a dairy farm [24]. The root mean square error (RMSE; Equation (3)) [31] was used as a measure of applicability:

(3) $$RMSE=\sqrt{\frac{1}{n}\times \sum^{\;}{\left(observed\;value-predicted\;value\right)}^{2}}$$

*n*: the total number of experimental values (values obtained from independent variables) or predicted values (values obtained from the developed survival model).

### 2.7. Development of Scenario from Market to Home

The exposure assessment scenario for the risk assessment of yogurt was divided into three stages: “market storage”, “transportation to home”, and “home storage”.

The storage temperature of yogurt in the market was investigated for an offline market, which was used as an input variable into an Excel (Microsoft@ Excel 2019, Microsoft Corp., USA) spreadsheet. PERT distribution was confirmed as the most suitable probability distribution model using @RISK 7.5 (Palisade Corp., Ithaca, NY, USA). The minimum, mode, and maximum values of storage temperature were 2.1, 7, and 9.7 °C, respectively. Storage time was also input based on the shelf-life of yogurt. The PERT distribution was confirmed as the most suitable model using @RISK 7.5 (Palisade Corp., Ithaca, NY. USA). The minimum, mode, and maximum values of storage time were 0, 240, and 312 h for drinking yogurt and 0, 240, and 480 h for regular yogurt, respectively.

At the stage of transporting from market to home, the pert distribution was applied to transportation time and temperature according to Jung [32]. Values of minimum (0.325 h, 10 °C), mode (0.984 h, 18 °C), and maximum (1.643 h, 25 °C) time and temperature were applied.

According to data from the MFDS [33], 69.2% of respondents answered that the most frequent storage period for milk was 2–3 days at the refrigeration temperature and the maximum storage period was 30 days or more. As a result, RiskPert (0, 60, 720 h) distribution was input in the scenario and a RiskLogLogistic (−10.407, 13.616, 8.611) distribution was used as the storage temperature [34].

### 2.8. Estimation of Consumption Data of Yogurt

The appropriate probability distribution model for consumption amount and intake rate of yogurt was confirmed using data from “Estimation of amount and frequency of consumption of 50 domestic livestock and processed livestock products” from the MFDS [35].

### 2.9. Hazard Characterization

For hazard characterization, the exponential model was used for the dose–response model of LM [36] (Equation (4)) and the Beta-Poisson model [37] was used for the dose–response model of EHEC (Equation (5)):

(4) p=1−exp(r × N)

P: the probability of foodborne illness for the intake of LM

r: the probability that one cell can cause disease (susceptible population: 1.06 ×10^−12^, general population: 2.37×10^−14^)

N: the number of cells exposed to the consumption of LM

(5) $$P=1-{\left(1+\frac{N}{\beta }\right)}^{-\alpha }$$

P: the probability of foodborne illness for the intake of EHEC

N: the consumption dose of EHEC

*α*: constant (0.49)

*β*: constant (1.81 × 10^5^)

### 2.10. Risk Characterization

To estimate the probability of foodborne illness per person per day for the intake of drinking and regular yogurt contaminated by LM or EHEC, formulas and inputs of exposure scenarios were written in an Excel spreadsheet. The risk was then calculated through a Monte Carlo simulation of @RISK. Median Latin hypercube sampling was used for sampling type, and a random method was used for generator seed. Finally, the correlation coefficient was calculated based on sensitivity analysis results to analyze factors affecting the probability of occurrence of foodborne illness.

### 2.11. Statistical Analysis

All experiments were repeated at least three times. All statistical analyses were performed using SAS version 9.4 (SAS Institute Inc., Cary, NC, USA). To describe significant variations of delta values between LM and EHEC at the same temperature, a t-test was used. Differences were considered significant at *p* < 0.05.

## 3. Results and Discussion

### 3.1. Prevalence and Intial Contamination Level in an On- an Offline Market

**Figure 1:**
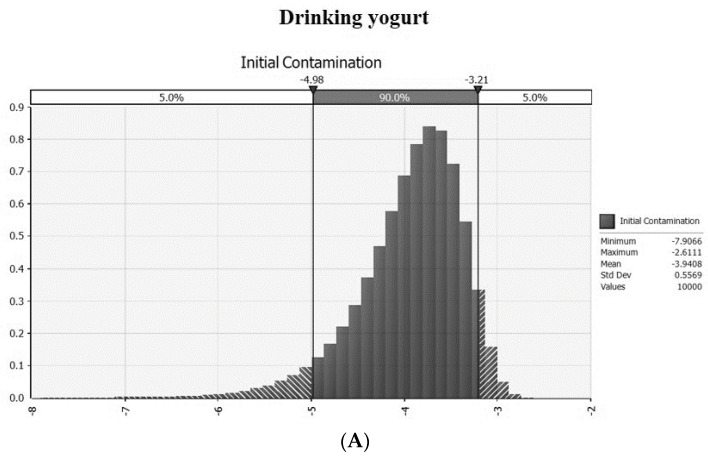

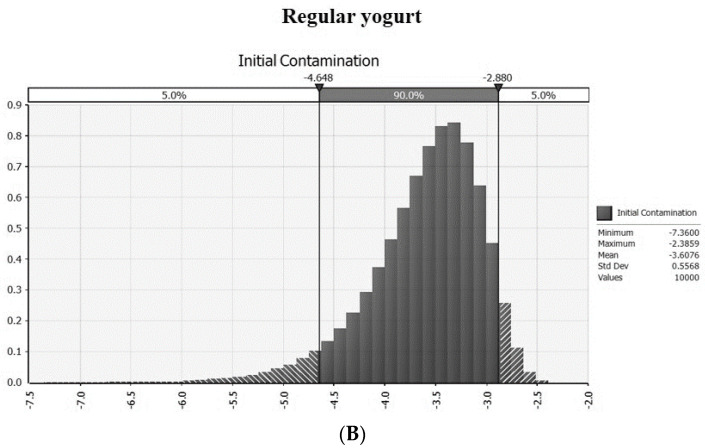
The probability distribution of initial contamination level of *Listeria monocytogenes* and EHEC in drinking (**A**) and regular yogurt (**B**).

As a first step in the exposure assessment, initial contamination levels for LM and EHEC were analyzed for drinking yogurt (*n* = 195) and regular yogurt (*n* = 90) purchased from on and offline markets in Korea. LM and EHEC were not detected in any samples [24]. The average contamination level was calculated using the equation [Log (−ln(1−PR)/weight)] by Sanaa et al. [27]. The average initial contamination level of both LM and EHEC was −3.941 log CFU/g in the drinking yogurt and −3.608 log CFU/g in the regular yogurt (Figure 1).

### 3.2. Development of Primary and Secondary Predictive Model

**Figure 2:**
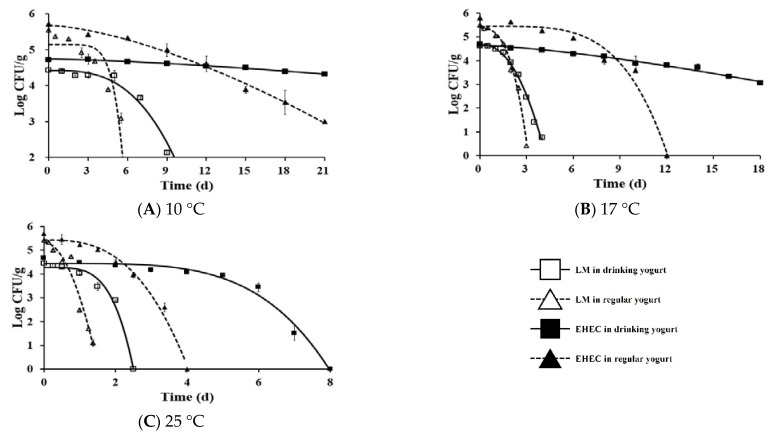
Primary survival models of *Listeria monocytogenes* (LM) and EHEC in yogurt as a function of temperature. LM in drinking yogurt: □, LM in regular yogurt: △, EHEC in drinking yogurt: ■, EHEC in regular yogurt: ▲.

**Figure 3:**
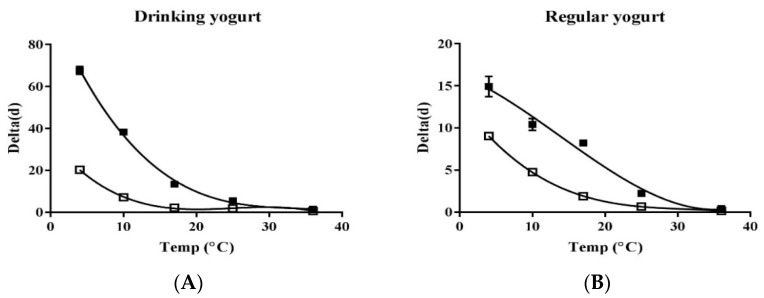
Secondary models for delta values of *Listeria monocytogenes* (□); and EHEC (■) in drinking (**A**) and regular yogurt (**B**).

The primary models of LM and EHEC in yogurt are shown in Figure 2. Secondary predictive models of delta values for LM and EHEC and equations are shown in Figure 3. Delta values of LM at 4, 10, 17, 25, and 36 °C were 20.31, 7.16, 2.15, 1.81, and 0.62 days in drinking yogurt and 9.04, 4.76, 1.89, 0.66, and 0.14 days in regular yogurt, respectively. Delta values of EHEC at 4, 10, 17, 25, and 36 °C were 67.61, 38.31, 13.42, 5.51, and 1.42 days in drinking yogurt and 14.93, 10.41, 8.21, 2.23, and 0.42 days in regular yogurt, respectively (Table 1). The delta value corresponds to the time for the first decimal reduction of the surviving populations of LM and EHEC. Overall, the higher the temperature, the lower the delta value, indicating that survival of LM and EHEC is better in yogurt stored at refrigeration temperature. Lactic acid bacteria (LAB) activity in yogurt increases as the temperature increases. Thus, the viability of LM and EHEC can be decreased. LAB can produce large amounts of organic acids and lower the pH value [38]. Some LAB can also produce bacteriocins and bacteriocin-like compounds to inhibit pathogens [39]. The temperature can affect the growth of LAB, and LAB isolated from Calabrian cheeses can inhibit the growth of LM in soft cheese [40]. LAB has the highest specific growth rate at 42–44 °C, the optimum growth temperature for LAB [41]. LAB starters can reduce the survival ability of EHEC in kimchi [42]. Bachrouri et al. [43] have reported that the viability of *E. coli* O157:H7 decreased as the temperature increased and *E. coli* O157:H7 is more resistant to death than nonpathogenic *E. coli* at 4 and 8 °C. The survival ability of LM is drastically decreased at 15 °C, but not significantly changed at 3~12 °C [44].

This work also noticed that LM and EHEC died faster in regular yogurt than in drinking yogurt due to the lower pH of regular yogurt (4.14 ± 0.02) than drinking yogurt (4.60 ± 0.02). This result is consistent with the study of Millet et al. [45], showing that low pH can decrease the growth of LM in raw-milk cheese. Guraya et al. [46] have also suggested that the viability of EHEC is drastically decreased in yogurt with pH below 4.1. Additionally, drinking yogurt has higher water activity (0.961 ± 0.001) than regular yogurt (0.943 ± 0.002) in this work. The Aw is the availability of the water in the product for microbes, and the higher the Aw, the better microorganism can survive. At 10 °C, the highest survival ability of EHEC was observed in drinking yogurt, followed by EHEC in regular yogurt, LM in drinking yogurt, and LM in regular yogurt (Figure 2). Overall, EHEC survived better than LM at especially low temperatures, regardless of the kind of yogurt in this work (Figure 3).

### 3.3. Validation

RMSE value is one of the parameters that can estimate the accuracy of the predictive model, and it was used to calculate the suitability of the model. The predictive model can be considered perfect if RMSE values are close to zero [47]. According to the study of model development using the Weibull model in heat-stressed *E. coli* O157:H7 and *L. monocytogenes* in kefir, RMSE values ranged from 0.13 to 0.52 in *E. coli* O157:H7 and 0.06 to 0.82 in *L. monocytogenes* [48]. The RMSE value calculated from the estimated data of LM was 0.185 in drinking yogurt and 0.115 in regular yogurt for interpolation. The RMSE value of EPEC was 1.079 in drinking yogurt and 1.001 in regular yogurt for extrapolation. As a result, the developed models in this study were judged to be appropriate to predict the survival of LM, EHEC, and EPEC in drinking and regular yogurt.

### 3.4. Change in Contamination Level of Listeria Monocytogenes and EHEC from Market to Home

The average contamination level of LM decreased −4.396 log CFU/g in drinking yogurt and −7.965 log CFU/g in regular yogurt at the market. The average contamination level of drinking yogurt during transportation from market to home was slightly decreased to −4.396 log CFU/g, and there was no change in regular yogurt. It was further decreased −5.00 log CFU/g for drinking yogurt and −10.25 log CFU/g for regular yogurt during storage at home before consumption.

The initial contamination level of EHEC was the same as that of LM. The contamination level of EHEC was −3.957 log CFU/g in drinking yogurt and −4.244 log CFU/g in regular yogurt at the market, which was maintained when yogurt was transported from market to home. The contamination level decreased −3.969 log CFU/g in drinking yogurt and −4.71 log CFU/g in regular yogurt before consumption at home. The contamination level of both LM and EHEC decreased in yogurt from the market to home because both pathogens cannot grow in yogurt, regardless of the type of yogurt. In this work, a more rapid decrease of contamination level of LM was observed than EHEC in regular yogurt.

Hu et al. [49] observed that organic acid produced from *Lactobacillus plantarum* isolated from traditional dairy products (kumis, milk thistle, yogurt) exhibits antimicrobial activity against pathogenic bacteria. They found that different proportions of organic acid (primarily lactic and acetic acid) show different antimicrobial activity against pathogenic bacteria. The difference in the proportion of organic acid between drinking and regular yogurt may affect the behavior of pathogens in yogurt.

### 3.5. Consumption Data of Yogurt

**Figure 4:**
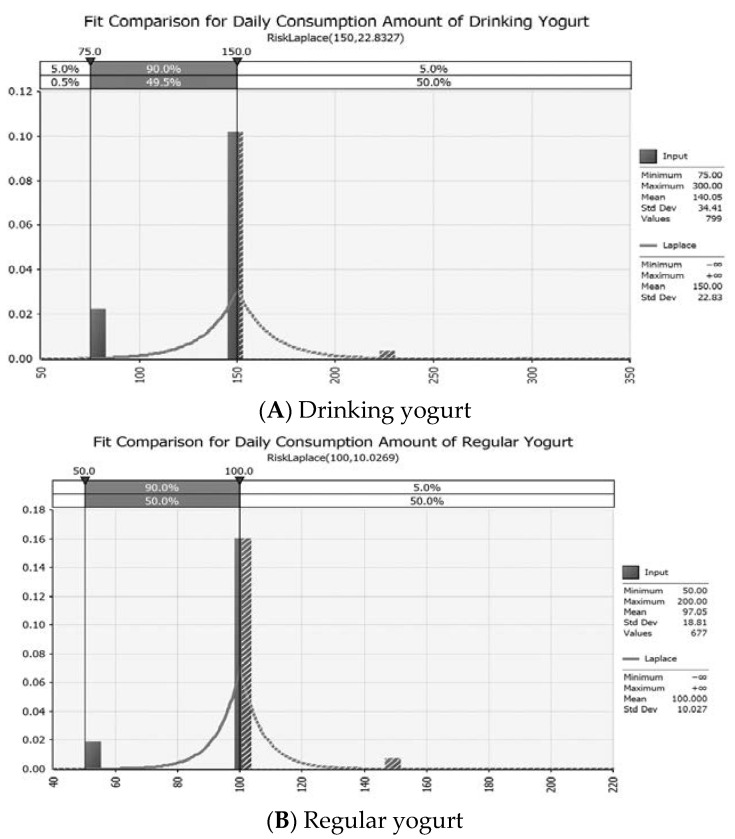
Probabilistic distribution for daily consumption amount of yogurt with @Risk.

The consumption amount and intake rate of yogurt are shown in Figure 4. As a result of fitting the distribution with @Risk, the RiskLaplace model was found to be the most suitable. Daily average consumption amounts of drinking yogurt and regular yogurt were 140 g and 97.046 g, respectively. Intake rates for drinking yogurt and regular yogurt were calculated to be 0.184 and 0.146, respectively. It could be concluded that the consumption of drinking yogurt was higher than that of regular yogurt.

### 3.6. Hazard Characterization and Risk Characterization

Final risks of LM and EHEC in yogurt were analyzed by separating susceptible population and general population using contamination level, consumption data, and dose–response model derived according to the scenario of the market to home (Table 2 and Table 3). As a result, no risk was estimated for the general group due to LM. However, the probability risk of foodborne illness due to LM was 1.91 × 10^−15^ in drinking yogurt and 2.87 × 10^−16^ in regular yogurt for susceptible populations per day. It is concluded that the risk of listeriosis is very low with yogurt consumption. The risk assessment result on LM in milk [36] demonstrates that the risk of milk consumption is also low (5.0 × 10^−9^ cases per serving).

By contrast, this was calculated to be 1.44 × 10^−8^ in drinking yogurt and 5.09 × 10^−9^ in regular yogurt with EHEC (Table 4). The risk of foodborne illness from both pathogens was higher from drinking yogurt due to its higher survival ability than regular yogurt. Additionally, the highest risk was found for EHEC in drinking yogurt due to the highest survival ability of EHEC in drinking yogurt (Figure 2), in which the highest delta value was noticed. As a result, the risk of EHEC is higher than LM in yogurt. Yogurt has an inhibition effect on pathogenic microorganisms due to organic acids such as lactic acid and acetic acid, which were produced by LAB [50], low pH below 4.1 [46], and bacteriocin or bacteriocin-like substances produced by LAB [51]. Yang et al. [51] isolated and identified bacteriocinogenic LAB from various cheeses and yogurts. They found that 20% of isolates (28 isolates) out of 138 LAB isolates had antimicrobial effects on all microorganisms tested, except for *E. coli.* In the present study, we found that EHEC shows better survival ability than LM in both types of yogurts. A similar trend was reported by Gulmez and Guven [52], who compared the inhibitory effects of LM, *E. coli* O157:H7, and *Yersinia enterocolitica* in yogurt and kefir samples during 24 h fermentation time and 10 days of storage. They found that *E. coli* O157:H7 showed the highest resistance during the yogurt’s fermentation and storage time. The most recent study showed [53] that most of the bacteriocins produced by LAB isolates are active against Gram-positive bacteria, such as LM and *Staphylococcus aureus,* whereas Gram-negative bacteria, *E. coli,* and *Salmonella* Typhimurium, displayed considerable resistance.

### 3.7. Sensitivity Analysis

**Figure 5:**
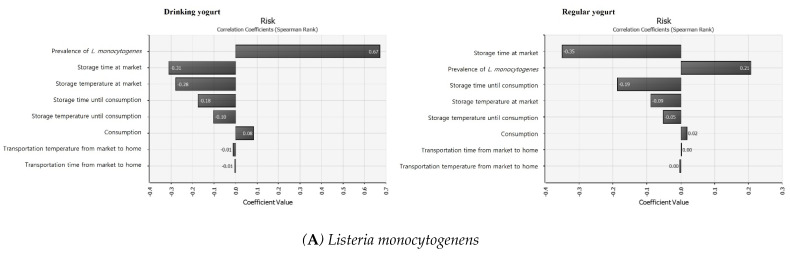

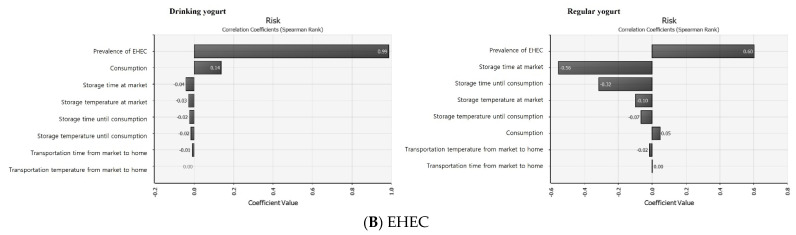
The correlation coefficient for sensitivity analysis affecting illness by *Listeria monocytogenes* (**A**) and EHEC (**B**) with consumption of yogurt with @Risk.

Sensitivity analysis was conducted to identify input variables with a major influence on results. If the result has a negative value, it has a negative correlation. As the input value increases, the output value decreases. If it is 0, there is no correlation. A positive value indicates a positive correlation, meaning that the output value increases as the input value increases [54]. Results of analysis of regression coefficients for the probability risk of foodborne illness caused by LM and EHEC due to yogurt consumption are shown in Figure 5. Both pathogens had a negative correlation with storage time at the market. The risk of foodborne illness decreased with increased storage time at the market. Both pathogens had the greatest positive correlation with the initial contamination level and consumption. As a result, it is considered that initial hygiene management before manufacture can reduce the risk of LM and EHEC. LM can survive longer in yogurt when LM is contaminated with higher concentrations during yogurt manufacture [55]. Kasımoğlu and Akgün [56] found that yogurt contaminated at 10^2^ CFU/g level of *E. coli* O157:H7 has a lower elimination time than that contaminated at 10^6^ CFU/g level. They suggested that the decline time of *E. coli* O157:H7 contaminated in the pre-fermentation stage could be affected by the initial contamination level. Therefore, initial hygiene management is important to inhibit the contamination and reduce the risk of pathogens in yogurt.

## 4. Conclusions

Results showed that the risk of serious illness from LM and EHEC due to drinking and regular yogurt consumption is very low. Yogurt does not permit the growth of LM and EHEC during storage at 4, 10, 17, 25, and 36 °C. The contamination level of both LM and EHEC decreased in yogurt from the market to home, and LM and EHEC died faster in regular yogurt than in drinking yogurt. However, controlling the initial contamination level of EHEC during yogurt manufacture should be emphasized because its survival ability in yogurt is higher in both drinking and regular yogurt than LM.

## Figures and Tables

**Table 1:** Survival kinetic parameters of *Listeria monocytogenes* (LM) and EHEC in yogurt ^1^.

Temperature (°C)	Pathogens	Drinking		Regular	
		Delta (day) ^2^	*p* ^3^	Delta (day)	*p*
4	LM ^4^	20.31 ± 0.20 *	0.73	9.04 ± 0.13 *	1.07 ± 0.07
	EHEC ^5^	67.61 ± 1.92 *	1.25 ± 0.01	14.93 ± 1.20 *	1.12 ± 0.06
10	LM	7.16 *	3.1 ± 0.08	4.76 ± 0.08 *	6.88 ± 0.44
	EHEC	38.31 ± 0.37 *	1.45 ± 0.01	10.41 ± 0.71 *	1.45 ± 0.12
17	LM	2.15 ± 0.01 *	2.27 ± 0.03	1.89 ± 0.06 *	3.32 ± 0.08
	EHEC	13.42 *	1.35 ± 0.08	8.21 ± 0.11 *	4.17 ± 0.16
25	LM	1.81 ± 0.03 *	4.43 ± 0.21	0.66 *	1.98 ± 0.02
	EHEC	5.51 ± 0.12 *	4.09 ± 0.21	2.23 ± 0.01 *	2.84 ± 0.03
36	LM	0.62 *	2.83 ± 0.03	0.14 *	1.17 ± 0.06
	EHEC	1.42 *	3.90 ± 0.05	0.42 ± 0.04 *	2.57 ± 0.33

^1^ Values are expressed as mean ± SD (*n* = 3). ^2^ Delta: Time for 1 log reduction. ^3^ *p*: Shape of graph. ^4^ LM: *Listeria monocytogenes.* ^5^ EHEC: Enterohemorrhagic *Escherichia coli.* * Significant difference of delta values was observed between LM and EHEC at the same temperature by t-test at *p* < 0.05.

**Table 2:** Simulation model and formulas in the Excel spreadsheet used to calculate the risk of *Listeria monocytogenes* (LM) in drinking and regular yogurt with @RISK.

Symbol	Unit	Definition	Formula	Reference
Product				
PR		Prevalence of LM in drinking yogurt	=RiskBeta(1, 196)	MFDS [24]
		Prevalence of LM in regular yogurt	=RiskBeta(1, 91)	
CL	CFU/g	Contamination level of LM	=−LN(1 − PR)/25	Sanna et al. [27]
IC	log CFU/g	Initial contamination level	=Log(CL)	
Market				
M_Time_	h	Storage time in market of drinking yogurt	=RiskPert(0, 240, 312)	MFDS [24]
		Storage time in market of regular yogurt	=RiskPert(0, 240, 480)	
M_Temp_	°C	Storage temperature in market	=RiskPert(2.1, 7, 9.7)	
Death				
Delta	h	Drinking yogurt	=823.8 + (−100.8) × M_Temp_ + 4.177 × M_Temp_^2^ + (−0.0556) × M_Temp_^3^	This research
		Regular yogurt	=315.1 + (−27.57) × M_Temp_ + 0.8396 × M_Temp_^2^ + (−0.0087) × M_Temp_^3^	
*p*		Drinking yogurt	=2.67 (Fixed)	
		Regular yogurt	=2.882 (Fixed)	
LM survival model	log CFU/g	C1	=IC − (M_Time_/delta)*^p^*	
Transportation to home				
T_Time_	h	Storage time during transportation	=RiskPert(0.325, 0.984, 1.643)	Jung [32]
T_Temp_	°C	Storage temperature during transportation	=RiskPert(10, 18, 25)	
Death				
Delta	h	Drinking yogurt	=823.8 + (−100.8) × T_Temp_ + 4.177 × T_Temp_^2^ + (−0.0556) × T_Temp_^3^	This research
		Regular yogurt	=315.1 + (−27.57) × T_Temp_ + 0.8396 × T_Temp_^2^ + (−0.0087) × T_Temp_^3^	
*p*		Drinking yogurt	=2.67 (Fixed)	
		Regular yogurt	=2.882 (Fixed)	
LM survival model	log CFU/g	C2	=C1-(T_Time_/delta)*^p^*	
Home				
H_Time_	h	Storage time until consumption	=RiskPert(0, 60, 720)	MFDS [33]
H_Temp_	°C	Storage temperature until consumption	=RiskLogLogistic(−10.407, 13.616, 8.611)	Bahk [34]
Death				
Delta	h	Drinking yogurt	=823.8 + (−100.8) × H_Temp_ + 4.177 × H_Temp_^2^ + (−0.0556) × H_Temp_^3^	This research
		Regular yogurt	=315.1 + (−27.57) × H_Temp_ + 0.8396 × H_Temp_^2^ + (−0.0087) × H_Temp_^3^	
*p*		Drinking yogurt	=2.67 (Fixed)	
		Regular yogurt	=2.882 (Fixed)	
LM survival model	log CFU/g	C3	=C2 − (H_Time_/delta)*^p^*	
Consumption				
Consume (Daily consumption average amount)		Drinking yogurt	=RiskLaplace(150, 22.833)	Park et al. [35]
		Regular yogurt	=RiskLaplace(100, 10.027)	
Intake rate(Distribution for consumption frequency)		Drinking yogurt	=0.184(Fixed)	
		Regular yogurt	=0.146(Fixed)	
Amount		Daily consumption average amount considered frequency	=Consume × Intake rate	
Dose-Response model				
Dose(D)		LM amount	=10^C3^ × Amount	
1-EXP(-r × D)		Parameter of r	=1.06 × 10^−12^ (Susceptible population)	FDA/WHO [36]
			=2.37 × 10^−14^ (General population)	
Risk Characterization				
Risk		Probability of illness/person/day	=1 − exp(−r × D)	FDA/WHO [36]

**Table 3:** Simulation model and formulas in the Excel spreadsheet used to calculate the risk of EHEC in drinking and regular yogurt with @RISK.

Symbol	Unit	Definition	Formula	Reference
Product				
PR		Prevalence of EHEC in drinking yogurt	=RiskBeta(1, 196)	MFDS [24]
		Prevalence of EHEC in regular yogurt	=RiskBeta(1, 91)	
CL	CFU/g	Contamination level of EHEC	=−LN(1 − PR)/25	Sanna et al. [27]
IC	log CFU/g	Initial contamination level	=Log(CL)	
Market				
M_Time_	h	Storage time in market of drinking yogurt	=RiskPert(0, 240, 312)	MFDS [24]
		Storage time in market of regular yogurt	=RiskPert(0, 240, 480)	
M_Temp_	°C	Storage temperature in market	=RiskPert(2.1, 7, 9.7)	
Death				
Delta	h	Drinking yogurt	=2347 + (−201.9) × M_Temp_ + 6.044 × M_Temp_^2^ + (−0.0616) × M_Temp_^3^	This research
		Regular yogurt	=391.7 + (−8.478) × M_Temp_ + (−0.4534) × M_Temp_^2^ + (−0.0109) × M_Temp_^3^	
*p*		Drinking yogurt	=2.406 (Fixed)	
		Regular yogurt	=2.429 (Fixed)	
EHEC survival model	log CFU/g	C1	=IC − (M_Time_/delta)*^p^*	
Transportation to home				
T_Time_	h	Storage time during transportation	=RiskPert(0.325, 0.984, 1.643)	Jung [32]
T_Temp_	°C	Storage temperature during transportation	=RiskPert(10, 18, 25)	
Death				
Delta	h	Drinking yogurt	=2347 + (−201.9) × T_Temp_ + 6.044 × T_Temp_^2^ + (−0.0616) × T_Temp_^3^	This research
		Regular yogurt	=391.7 + (−8.478) × T_Temp_ + (−0.4534) × T_Temp_^2^ + (−0.0109) × T_Temp_^3^	
*p*		Drinking yogurt	=2.406 (Fixed)	
		Regular yogurt	=2.429 (Fixed)	
EHEC survival model	log CFU/g	C2	=C1 − (T_Time_/delta)*^p^*	
Home				
H_Time_	h	Storage time until consumption	=RiskPert(0, 60, 720)	MFDS [33]
H_Temp_	°C	Storage temperature until consumption	=RiskLogLogistic(−10.407, 13.616, 8.611)	Bahk [34]
Death				
Delta	h	Drinking yogurt	=2347 + (−201.9) × H_Temp_ + 6.044 × H_Temp_^2^ + (−0.0616) × H_Temp_^3^	This research
		Regular yogurt	=391.7 + (−8.478) × H_Temp_ + (−0.4534) × H_Temp_^2^ + (−0.0109) × H_Temp_^3^	
*p*		Drinking yogurt	=2.406 (Fixed)	
		Regular yogurt	=2.429 (Fixed)	
EHEC survival model	log CFU/g	C3	=C2 − (H_Time_/delta)*^p^*	
Consumption				
Consume (Daily consumption average amount)		Drinking yogurt	=RiskLaplace(150, 22.833)	Park et al. [35]
		Regular yogurt	=RiskLaplace(100, 10.027)	
Intake rate(Distribution for consumption frequency)		Drinking yogurt	=0.184(Fixed)	
		Regular yogurt	=0.146(Fixed)	
Amount		Daily consumption average amount considered frequency	=Consume × Intake rate	
Dose-Response model				
Dose(D)		EHEC amount	=10^C3^ × Amount	
Model		Parameter of α	=0.49	Park et al. [37]
		Parameter of β	=1.81 × 10^5^	
Risk characterization				
Risk		Probability of illness/person/day	=1 − (1 + D/β)^−α^	Park et al. [37]

**Table 4:** Probability of illness per day per person by *Listeria monocytogenes* (LM) and EHEC with consumption of yogurt with @Risk scenario.

Probability of Illness/Person/Day							
Pathogens	Sample		Min	25%	Mean	95%	Max
LM	Drinking	Susceptible population	0	0	1.91 × 10^−15^	8.44 × 10^−15^	3.65 × 10^−14^
		General population	0	0	0	0	0
	Regular	Susceptible population	0	0	2.87 × 10^−16^	2.11 × 10^−15^	3.63 × 10^−14^
		General population	0	0	0	0	0
EHEC	Drinking		0	4.01 × 10^−9^	1.44 × 10^−8^	4.33 × 10^−8^	1.75 × 10^−7^
	Regular		0	4.39 × 10^−10^	5.09 × 10^−9^	2.12 × 10^−8^	9.45 × 10^−8^

## Data Availability

We did not report any additional data for this study.
